# Precise Control of the Number of Layers of Graphene by Picosecond Laser Thinning

**DOI:** 10.1038/srep11662

**Published:** 2015-06-26

**Authors:** Zhe Lin, Xiaohui Ye, Jinpeng Han, Qiao Chen, Peixun Fan, Hongjun Zhang, Dan Xie, Hongwei Zhu, Minlin Zhong

**Affiliations:** 1 School of Materials Science and Engineering, Tsinghua University, Beijing 100084, China; 2 State Key Laboratory of New Ceramics and Fine Processing, Tsinghua University, Beijing 100084, China; 3 Tsinghua National Laboratory for Information Science and Technology (TNList), Institute of Microelectronics, Tsinghua University, Beijing 100084, China

## Abstract

The properties of graphene can vary as a function of the number of layers (NOL). Controlling the NOL in large area graphene is still challenging. In this work, we demonstrate a picosecond (ps) laser thinning removal of graphene layers from multi-layered graphene to obtain desired NOL when appropriate pulse threshold energy is adopted. The thinning process is conducted in atmosphere without any coating and it is applicable for graphene films on arbitrary substrates. This method provides many advantages such as one-step process, non-contact operation, substrate and environment-friendly, and patternable, which will enable its potential applications in the manufacturing of graphene-based electronic devices.

Graphene, a one-atom thick layer of graphite with densely packed sp^2^-bonded carbon atoms^1^, has been extensively investigated since its first production/generation in the lab by Geim, *et al.*^2^ in 2004. The unique properties such as electron mobility of 2.5 × 10^5^ cm^2^V^−1^s^−1^ at room temperature^3^, high thermal conductivity (>3000 WmK^−1^)^4^,^5^ and optical absorption of exactly πα = 2.3% have been reported^6^. Depending on these properties, graphene is considered to be the next-generation materials of transistors, transparent conductive electrodes and in other applications^7^. The properties of graphene, such as electronic structure and optical transparency are functions of its number of layers (NOL)^8^,^9^. Therefore, it is significantly important to accurately control the number of graphene layers. Although many groups have reported the synthesizing of monolayer or bilayer graphene, to control the NOL in graphene synthesis is still challenging^10^. Thus, the post-treatment of few-layer graphene to achieve the accurate control of the NOL is an alternative approach.

There are several studies which reported the top-down methods that used some methods reducing graphene thickness to achieve the specific number of layers. Huang, *et al.*^11^ reported an *in-situ* sublimation of the suspended graphene inside transmission electron microscopy (TEM). Graphene was peeled layer-by-layer using Joule heating to induce the atom sublimation and electron beam irradiation to facilitate the atom displacement. Yang, *et al.*^7^ proposed a simple and efficient method of thinning graphene by mild nitrogen plasma irradiation and annealing in Ar/O_2_. Nitrogen plasma attacked the top-layer plane, introducing defects into the lattice; oxygen molecules etched out the carbon atoms at the edge of defect sites, finally removing the damaged top layer. All other top-down methods also included inducing the defects on the top layers of graphene through oxidation or plasma treatments^7^,^12^. Han, *et al.*^13^ proposed a method of producing monolayer graphene by laser irradiation. The upper layers of graphene were selectively oxidized under the laser heating; and the bottom monolayers of graphene were kept well because the substrates played as a heat sink and absorbed the extra energy. Dimiev, *et al.*^8^ demonstrated a novel approach of layer-by-layer removal of graphene. A Zn layer was first coated on the few-layer graphene by sputtering. Then Zn was dissolved in diluted acid, meanwhile, the acid removed the damaged graphene top layers. All of the methods mentioned above showed the capability of thinning graphene, and some had a good potential in accurately controlling the number of graphene layers. However, it is obvious that all these methods have some kinds of limitations. The layer-by-layer thinning method includes making defects on the graphene top layers and removing the damaged layers, followed by a multiple-step process, which requires more equipment and strict reaction/processing conditions. Furthermore, these methods can only remove one graphene layer in one process, which represents that if a few layers of graphene are needed to be removed, the process needs to be repeated several times. On the other hand, the one-step methods can only thin the graphene to monolayer or bilayer, which cannot reach the purpose of preparing electronics with different properties by controlling the number of graphene layers.

Laser has been employed in graphene treatments, including direct laser writing, laser patterning and laser induced synthesis. For examples, EI-Kady, *et al.*^14^ reported a graphene-based electrochemical capacitor with reduced graphene oxide electrodes obtained using a standard light-scribe DVD laser. Kalita, *et al.*^15^ proposed a method for micropatterning of graphene by femtosecond laser. Park, *et al.*^16^ demonstrated a rapid single-step fabrication of graphene patterns using laser assisted chemical vapor deposition (LCVD).

Here, a novel yet simple method of picosecond (ps) laser thinning is proposed to accurately control the number of graphene layers. The graphene films can be thinned to the specific number of layers using the scanning laser radiation with tunable pulse threshold energy in atmospheric condition. In the process, arbitrary patterns are achievable if the unique scanning paths are set up. Because this process/method has many advantages, such as one-step process, non-contact operation, substrate and environmental-friendly, and patternable thinning, it will be potentially used in the fabrication of graphene-based electronic devices.

## Results

### Laser Thinning

**Figure 1:**
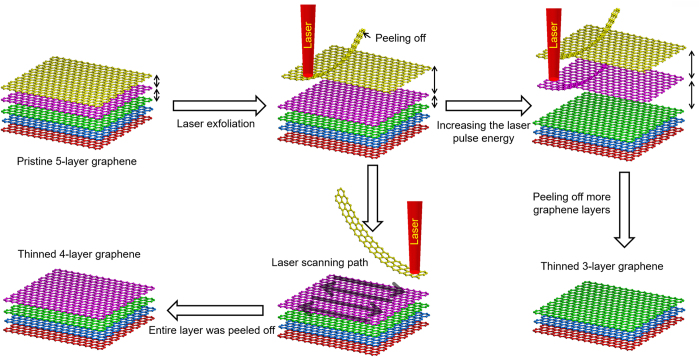
Schematics of laser thinning process.

The ps laser thinning process of graphene is demonstrated in Fig. 1. The laser irradiated on the pristine graphene sample (5-layer), causing the increasing of the distance between the top layer and the bottom layers. Then the graphene within the laser irradiation area was peeled off. The whole layer was peeled off by the laser beam scanning and the graphene sample was thinned to 4-layer. Furthermore, by increasing the laser pulse energy, more top layers were lifted up and peeled off from bottom, to obtain graphene film with less number of layers. Therefore, the number of graphene layers can be precisely controlled by adjusting the laser pulse energy.

**Figure 2:**
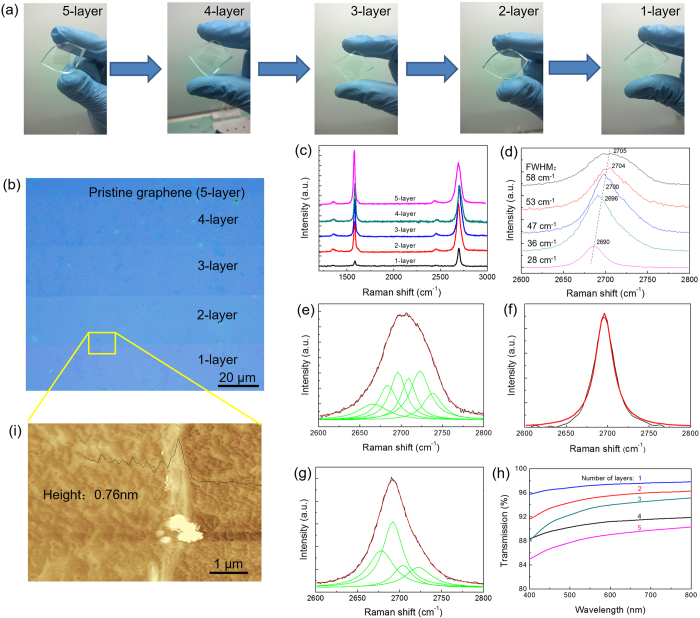
(**a**) Photographs and (**b**) optical image of graphene with different NOL obtained by laser thinning. (**c**) Raman spectra of graphene with different numbers of layers. (**d**) Corresponding 2D peak position and FWHM. The splitting of 2D peak by Lorentzian fitting: (**e**) 5-layer, (**f**) monolayer and (**g**) bilayer graphene. (**h**) Transmittance spectra. (**i**) AFM image.

Figure 2a shows the photographs of graphene transferred on the transparent substrate, *e.g.* poly(dimethylsiloxane)(PDMS). The pristine (5-layer) to monolayer graphene are displayed from left to right. A typical optical image of graphene with different layer numbers is shown in Fig. 2b. The contrast with graphene and substrate (SiO_2_/Si) indicates the number of layers, which is further confirmed by Raman (Fig. 2c–g), visible transmission spectra (Fig. 2h) and Atomic Force Microscopy (AFM) image of graphene (Fig. 2i).

Raman is a powerful tool to determine the number of graphene layers and evaluate their qualities^7^. The two intense peaks located at ~1580 cm^−1^ and ~2700 cm^−1^ represent the characteristic G peak and 2D peak. The peak intensity ratio of G to 2D (*I*_G_/*I*_2D_) reflects the number of graphene layers. The position, full width at half maximum (FWHM), and Lorentzian fitting of 2D peak supply further evidences for determining the layer number of graphene^15^. Raman spectra in Fig. 2c were recorded from the samples as shown in Fig. 2b from top to bottom. The decrease of *I*_G_/*I*_2D_ from 1.3 to 0.3 (Fig. 2c) reveals the reduction of graphene layers from 5 to 1. In Fig. 2d, the position of 2D peak shows blue-shifts from 2705 cm^−1^ (5-layer) to 2690 cm^−1^ (1-layer) and the FWHM (2D) decreases from 58 cm^−1^ (5-layer) to 28 cm^−1^ (1-layer), which is consistent with the result that the 2D peak position bluely shift following the layer decrease^15^. For a multilayer graphene, the 2D peak is asymmetric and composed of multiple sub-peaks, as shown in Fig. 2e. However, upon laser thinning, the monolayer graphene shows a symmetric 2D peak, which is fitted by only one sub-peak (Fig. 2f). Meanwhile, the bilayer graphene has a symmetric 2D peak and can be fitted by four sub-peaks (Fig. 2g). All these results indicate that the graphene film is indeed thinned through the laser thinning process.

To accurately determine the number of graphene layers, the visible transmittances of graphene samples were measured. The optical image shows that the graphene films are uniform after the same laser thinning process, and the transmittance of graphene monotonically decreases following the layer increase of graphene. As shown in Fig. 2h, the transmittance of monolayer graphene is 97.22%, which is well fitted with the theoretical value^1^ of the optical absorption (2.3%) for monolayer graphene. With the layers increasing one by one, the transmittance decreases correspondingly from 97.22% to 88.60%, and the pristine graphene with 5 layers has the lowest transmittance. Further, the AFM image in Fig. 2i correlates the position of the yellow box in Fig. 2b. The relative height difference between the bilayer and monolayer is 0.76 nm, which is conformed to the height of 1-layer graphene prepared by CVD method^8^. Therefore, pristine graphene films of 5-layer are thinned by the laser to 4-layer, 3-layer, bilayer and monolayer graphene, separately. The resistance of pristine graphene was about 200 Ω, which was increased to about 500 Ω when the number of layers number was reduced to 1. It is worth noting that the oxidation and doping could influence the surface resistance. Raman spectra of both thinned areas and peeled-off pieces showed that there were few oxidation sates present. Therefore, we considered the change of surface resistance was mainly affected by the number of layers of graphene.

### Laser Patterning

**Figure 3:**
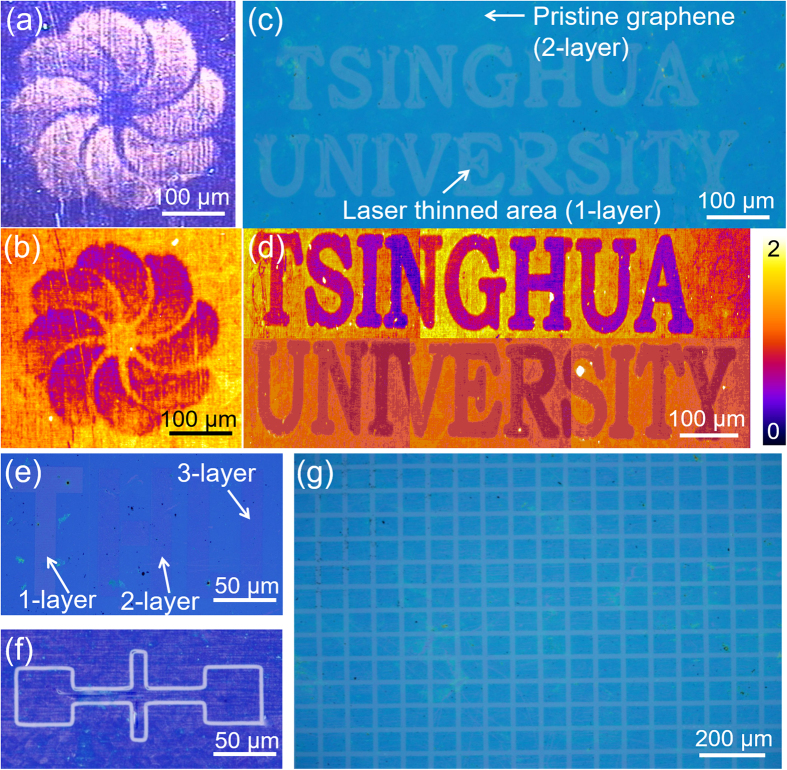
(**a**) Optical image and (**b**) Raman mapping image of a windmill pattern. (**c**) Optical image and (**d**) Raman mapping image of the word pattern “TSINGHUA UNIVERSITY”. (**e**) Optical contrast of graphene layers obtained with different laser thinning parameters. The letter “T” represents the monolayer, “H” bilayer, and “U” tri-layer graphene. (**f**) FET-like and (**g**) mesh-like patterns of graphene.

The results reported above demonstrate that the one-step laser processing method can produce the graphene with accurate NOL. On the other hand, since graphene-based materials are considered as the promising alternatives to silicon-based ones in the future electronic devices, the patterning of graphene is an essential step in microelectronic processing^17^. Figure 3a shows a windmill pattern produced by laser on graphene sample, in which the windmill area is monolayer graphene and the pristine area is 5-layer. The result is confirmed by the Raman mapping image recorded at the same area (Fig. 3b). The contrast in the Raman image indicates the different radios of *I*_G_/*I*_2D_. The purple area represents the lowest *I*_G_/*I*_2D_ radio, which indicates the presence of monolayer graphene. The yellow area represents the 5-layer graphene. The word of “TSINGHUA UNIVERSITY” with monolayer graphene is patterned on a bilayer graphene sample (Fig. 3c) and the Raman image from the same area is shown in Fig. 3d. The background radio of *I*_G_/*I*_2D_ is about 1, which points out that the pristine graphene is bi-layered. After the laser processing, the letter area is thinned to monolayer, which is determined by the *I*_G_/*I*_2D_ radio reduced to 0.5. Using different laser energy density, graphene samples with different NOL are obtained. The areas of letter T, H and U are monolayer, bilayer and 3-layer graphene, respectively.

## Discussion

### Mechanism

**Figure 4:**
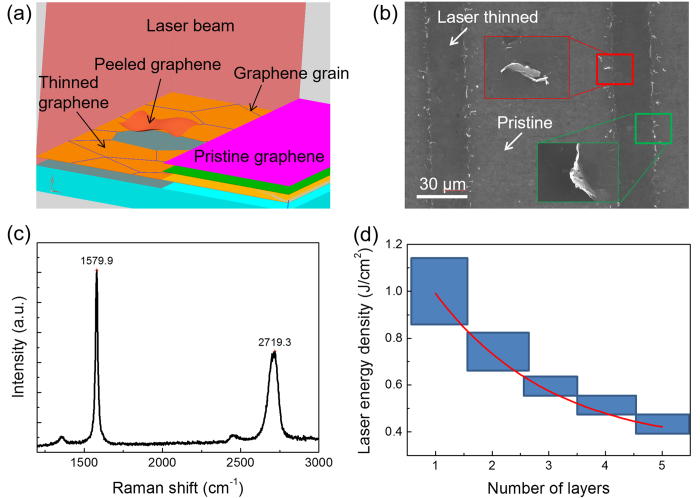
(**a**) Schematics for the mechanism of laser thinning. (**b**) SEM image and (**c**) Raman spectrum of the graphene sample after laser peeling off. (**d**) Thresholds of the laser energy density to obtain graphene with different NOL.

The mechanism of the one-step laser thinning process is further proposed. Figure 4a shows the schematic of the “peeling off” mechanism of graphene thinning. The D peak at ~1350 cm^−1^ represents the signal from defects and impurities, and the ratio of D peak and G peak (*I*_D_/*I*_G_) is widely applied to characterize the crystallization quality of graphene^18^. Figure 2c reveals that the *I*_D_/*I*_G_ keeps fairly low during all the thinning process, which is different from the layer- by-layer methods^6^. The low D peak indicates that there is no oxidation during the thinning process. The photon energy of the laser used in this process was 1.2 eV (1064 nm laser), which was much lower than the formation energy of C-C bond in ideal graphene (3.7 eV). The laser couldn’t directly break the C-C bond and sublimate the graphene. However, the graphene samples used in our experiments were grown using CVD method, and the graphene layers were composed of grains containing crystalline defects. The bonds on the grain boundaries with defects were much weaker than the defect-free C-C bonds. These defect bonds could be easily broken by the laser photons. The Van der Waals force, which attracted the neighboring graphene layers together, was 0.17 eV, much smaller than the photon energy of the laser. In the interaction of the photon and graphene, the distance of the interacting grains and their adjacent layers increased^17^, the Van der Waals force was weakened further, and finally the grains lost the connection with adjacent grains and were peeled off from underlying graphene layers.

Scanning electron microscope (SEM) was utilized to prove this layer thinning mechanism. Figure 4b shows the SEM image of the laser thinned and pristine areas. To obtain more details about the peeling off mechanism, the thinning process was conducted at a higher scanning speed (200 mm/s). The residues at the scanning boundary were acquired which did not exist under the optimized scanning parameters. The curls of graphene emerged on the scanning boundaries, *i.e.*, graphene grain residues which were not being completely peeled off (marked with the green box in Fig. 4b). However, the graphene grains in the middle area of the scanning were completely peeled off. Most of grains peeled off by laser disappeared in the air, while some pieces of graphene grains fell onto the graphene near the scanning boundaries (marked with the red box in Fig. 4b). The grain size was about 1 μm, which was much smaller than the laser spot diameter. The graphene sample after being peeled off (Fig. 4b) was characterized by Raman (Fig. 4c). The *I*_G_/*I*_2D_ is about 2, much higher than the pristine graphene, which indicates the graphene in this area is deformed. It is worth noting that the graphene after being peeled off still has a quite low D peak, which further proves that no oxidation occurs in this process.

The threshold of the laser energy density to thin graphene into specific layers is summarized in Fig. 4d. To obtain monolayer graphene from pristine 5-layer graphene, the threshold of the laser energy density should be up to 1.0 J/cm^2^, which is much higher than the threshold for obtaining a 4-layer graphene (0.5 J/cm^2^). Dhar, *et al.*^19^ studied the relationship of the ablation threshold energy and the numbers of remaining graphene layers. The ablation threshold decreased dramatically following the increase of obtained graphene layers. These phenomena were explained by the different optical absorption coefficients and specific heat of graphene with different layers. This theory explained why laser photons with different energy density could peel off different graphene layers within one process. For example, to get 4-layer graphene, the specific energy density of applied laser photons was higher than the ablation threshold of 4-layer graphene, but lower than that of 3-layer graphene. So only the top layer was peeled off, and the second top-layer was retained. The window of the two adjacent ablation thresholds is the applied energy range in the laser thinning to obtain the graphene with specific layers. This makes obtaining graphene with specific number of layers in one step is achievable. These thresholds were obtained when the grain size of graphene is about 1 μm. Based on the peeling off mechanism proposed above, it can be predicted that the threshold energy will increase with the grain size.

### Devices

Due to the unique thickness-dependent electronic properties, high flexibility, and transparency, graphene and its relatives are fantastic materials for both electron active channels and electrodes in various electronic devices^20^. Graphene with different NOL have different electronic properties. To demonstrate this, field-effect transistors (FETs) were fabricated, as shown in Fig. S1. First, pristine graphene was transferred onto a silicon substrate with 90 nm SiO_2_. Then, graphene was thinned to the accurate NOL by laser thinning. As the channel materials and the electrodes, graphene was patterned by lithography, which is displayed in the top-right inset of Fig. S1. The field-effect characteristics with different layer graphene were tested. The mobility of monolayer graphene FET is about 1.4 times of that for bilayer graphene FET. When the NOL is increasing up to 5, the mobility significantly drops to only 130 cm^2^/Vs. Nagashio, *et al.*^21^,^22^ reported that with decreasing NOL, the current modulation was enhanced because of the reduction of the interlayer scattering. On the other hand, when the NOL decreased from the bilayer to monolayer, the mobility drastically increased due to the inherent change from the quadric to linear dispersion. While with the increase in NOL, the mobility decreased slightly because of the interlayer scattering. Our result is consistent with the theory, which further confirms that the laser thinning method can accurately control the number of graphene layers.

## Conclusion

To conclude, a one-step laser thinning process, with features of non-contact operation, substrate and environment-friendly and patternable, is demonstrated to obtain the accurate number of graphene layers. A “peeling off” mechanism of the laser process is proposed. The thinning process is conducted in atmosphere without any coating. Besides, the laser scanning is applicable for graphene films on arbitrary substrates. All of these advantages make this method have broad potential applications in graphene-based electronic devices.

## Experimental Section

### Preparation and laser treatment of grapheme

Pristine graphene samples were prepared by CVD on copper substrates. The numbers of layers of as-prepared graphene films were confirmed to be 5 by optical microscopy, Raman spectroscopy and optical absorption. Graphene films were then transferred onto Si/SiO_2_ substrates^1^. The laser using in this work was in picosecond scale with the power of 100 W, and the frequency of 2 MHz. The pattern setup was controlled by a galvanometer, which made the laser beam moving as sited on an objective and with a focus spot of 30 μm. The scanning speed of laser was 100 mm/s, corresponding to a trimming speed of 180 mm^2^/min. The whole process was conducted in the air ambient.

### Characterizations

The optical images were taken using a Nikon microscope. SEM images were obtained using a LEO-1530 microscope (LEO Electron Microscopy. GERMANY). Raman spectra were collected with a HORIBA Lab RAM HR spectrometer (514 nm laser excitation) (HORIBA Jobin Yvon. FRANCE). Raman mapping images were recorded by a Raman 11 spectroscopy (Nanophonon. JAPAN). AFM images were acquired by a SPM-960 instrument (SHIMADZU. JAPAN). The transmission spectra of graphene were obtained with a UV-visible spectrophotometer.

## Additional Information

**How to cite this article**: Lin, Z. *et al.* Precise Control of the Number of Layers of Graphene by Picosecond Laser Thinning. *Sci. Rep.* **5**, 11662; doi: 10.1038/srep11662 (2015).

## Supplementary Material

Supplementary Information
