# Supplementary material for: Precise Control of the Number of Layers of Graphene by Picosecond Laser Thinning
Source: Sci Rep. 2015 Jun 26;5:11662. doi: 10.1038/srep11662 (PMC4481519; doi:10.1038/srep11662)
Supplement: Supplementary Information [file srep11662-s1.pdf]

Supplementary Information

Precise Control of the Number of Layers of Graphene by Picosecond Laser Thinning

Zhe Lin[1], Xiaohui Ye[1], Jinpeng Han[1], Qiao Chen[1,2], Peixun Fan[1], Hongjun Zhang[1], Dan Xie[3], Hongwei Zhu[1,2*], Minlin Zhong[1*]

[1]School of Materials Science and Engineering, Tsinghua University, Beijing 100084, China

[2]State Key Laboratory of New Ceramics and Fine Processing, Tsinghua University, Beijing 100084, China

[3]Tsinghua National Laboratory for Information Science and Technology (TNList), Institute of Microelectronics, Tsinghua University, Beijing 100084, China

[*]Emails: zhml@tsinghua.edu.cn, hongweizhu@tsinghua.edu.cn

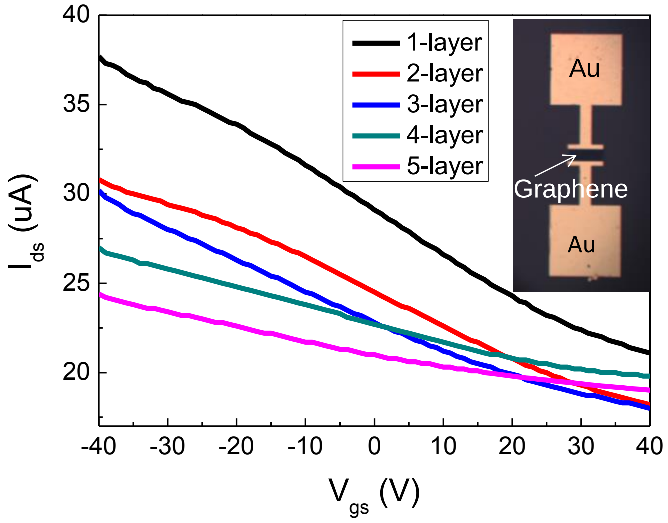

Figure S1. Source-drain current *vs.* gate voltage plots from graphene FETs of different number of layers. The channel length is 30 μm. The inset shows the optical image of a FET device.
